# Incidence, risk factors, and impact on mortality of status epilepticus in sepsis in the United States

**DOI:** 10.1186/cc9748

**Published:** 2011-03-11

**Authors:** J Urtecho, A Seifi, M Maltenfort, M Vibbert, W McBride, M Moussouttas, J Jallo, R Bell, F Rincon

**Affiliations:** 1 Thomas Jefferson University, Philadelphia, PA, USA

## Introduction

We sought to determine the epidemiology of status epilepticus (SE), prevalence of risk factors and impact on hospital mortality in sepsis in the United States. We hypothesized that SE would be associated with increased mortality.

## Methods

Data were derived from the National Inpatient Sample from 1998 to 2008. We included patients older than 18 years, with a primary diagnosis of sepsis and SE. Definitions were based on the International Classification of Diseases, Ninth Revision, Clinical Modification Codes (ICD-9). Adjusted incidence rates, prevalence odds ratios (ORs) and 95% confidence intervals (CIs) were calculated. Multivariate logistical models assessed for the impact of SE on hospital mortality.

## Results

**Figure 1:**
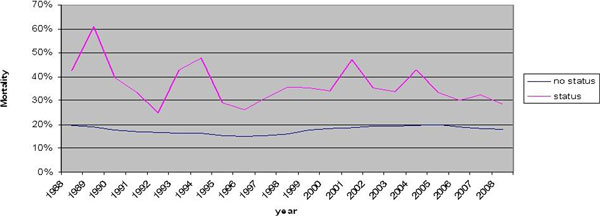
**In-hospital mortality in septic patients**.

We identified 7,672,551 admissions with diagnosis of sepsis and 7,619 with SE from 1998 to 2008. The population-adjusted rate of sepsis increased from 72/100,000 in 1998 to 250/100,000 in 2008. In septic patients, SE was more common in older patients, in women than men, in urban academic centers than rural centers, in those with respiratory dysfunction and metabolic dysfunction. Total in-hospital mortality fell from 20% in 1998 to 18.1% in 2008, yet the number of deaths increased over the study period. Mortality was highest among SE (OR = 1.7; 95% CI = 1.4 to 1.9) (Figure 1), older patients, men, those with respiratory dysfunction, cardiovascular dysfunction, hematologic dysfunction, metabolic dysfunction, renal dysfunction and hepatic encephalopathy.

## Conclusions

Our study demonstrates the incidence of SE in sepsis is increasing. Despite a decline in sepsis-related mortality, the presence of SE doubles the risk of in-hospital death. Further study is needed to determine whether detection and treatment of SE will impact mortality.
